# Effect of Alcoholic Intoxication on the Risk of Inflammatory Bowel Disease: A Nationwide Retrospective Cohort Study

**DOI:** 10.1371/journal.pone.0165411

**Published:** 2016-11-01

**Authors:** Tai-Yi Hsu, Hong-Mo Shih, Yu-Chiao Wang, Leng-Chieh Lin, Guan-Yi He, Chih-Yu Chen, Chia-Hung Kao, Chao-Hsien Chen, Wei-Kung Chen, Tse-Yen Yang

**Affiliations:** 1 Department of Emergency Medicine, China Medical University Hospital, Taichung, Taiwan; 2 School of Medicine, College of Medicine, China Medical University, Taichung, Taiwan; 3 Management Office for Health Data, China Medical University Hospital, Taichung, Taiwan; 4 Department of Emergency Medicine, Chang Gung Memorial Hospital, Chiayi, Taiwan; 5 Department of Nursing, Chang Gung University of Science and Technology, Chiayi, Taiwan; 6 Graduate Institute of Clinical Medicine, National Taiwan University College of Medicine, Taipei, Taiwan; 7 Department of Dermatology, National Taiwan University Hospital Yunlin Branch, Douliou, Taiwan; 8 Graduate Institute of Clinical Medical Science and School of Medicine, College of Medicine, China Medical University, Taichung, Taiwan; 9 Department of Nuclear Medicine and PET Center, China Medical University Hospital, Taichung, Taiwan; 10 Department of Bioinformatics and Medical Engineering, Asia University, Taichung, Taiwan; 11 Department of Medical Laboratory Science and Biotechnology, China Medical University, Taichung, Taiwan; 12 Department of Medical Research, China Medical University Hospital, China Medical University, Taichung, Taiwan; Case Western Reserve University, UNITED STATES

## Abstract

**Purpose:**

This study investigated whether alcoholic intoxication (AI) increases the risk of inflammatory bowel disease (IBD) by using a population-based database in Taiwan.

**Methods:**

This retrospective matched-cohort study included 57 611 inpatients with new-onset AI (AI cohort) and 230 444 randomly selected controls (non-AI cohort). Each patient was monitored for 10 years to individually identify those who were subsequently diagnosed with Crohn disease (CD) and ulcerative colitis (UC) during the follow-up period. Cox proportional hazard regression analysis was conducted to determine the risk of IBD in patients with AI compared with controls without AI.

**Results:**

The incidence rate of IBD during the 10-year follow-up period was 2.69 per 1 000 person-years and 0.49 per 1 000 person-years in the AI and non-AI cohorts, respectively. After adjustment for age, sex, and comorbidity, the AI cohort exhibited a 3.17-fold increased risk of IBD compared with the non-AI cohort (hazard ratio [HR] = 3.17, 95% confidence interval [CI] = 2.19–4.58). Compared with the non-AI cohort, the HRs of CD and UC were 4.40 and 2.33 for the AI cohort, respectively. After stratification for the severity of AI according to the duration of hospital stay, the adjusted HRs exhibited a significant correlation with the severity; the HRs of IBD were 1.76, 6.83, and 19.9 for patients with mild, moderate, and severe AI, respectively (*p* for the trend < .0001).

**Conclusion:**

The risk of IBD was higher in patients with AI and increased with the length of hospital stay.

## Introduction

Inflammatory bowel disease (IBD), including Crohn disease (CD) and ulcerative colitis (UC), is a chronic relapsing inflammatory disorder of the intestine with unknown causes. The etiology of IBD is described as involving interactions among microbial, genetic, environmental, and immunological factors [1]. The previous study mentioned that the alcohol intoxication impairs the intestinal barrier bio-function, which might enhance the microbial toxins translocation, thereby contributing to inflammatory status in alcoholic gastrointestinal (GI) diseases [2, 3].

Long-term alcohol consumption is one of the possible latent risk factors, especially in severe alcohol consumption, like alcohol abuse and/or intoxication, is one of the possible surrogate markers, but no consensus about the effect of alcohol on IBD has been well established. Alcoholic intoxication, or hazardous and harmful alcohol use, can cause major GI diseases such as gastritis, cirrhosis, hepatitis, and pancreatitis [4]. Little is known regarding the relationship between inflammatory bowel diseases and alcohol intoxication in a population based approach.

The understanding of the association between alcoholic intoxication and IBD will facilitate preventing and treating IBD. Therefore, we conducted a retrospective cohort study to interpret 1) whether the IBD risk are apparently risen among patients with various alcoholic intoxication severity, and 2) whether this risk for IBD is revealed in the presence of existing chronic illness.

## Materials and Methods

The Preferred Statements were followed the reporting items of the REporting of studies Conducted using Observational Routinely collected health Data (RECORD) checklist [5]. The RECORD checklist of observational studies which extended from STROBE statement are available as supporting information (S1 Checklist).

### Data Source

Data were collected from the National Health Insurance Research Database (NHIRD), a database containing comprehensive claims data collected from the Taiwan National Health Insurance (NHI) program. This program was introduced in 1995 and covered over 99% of the 23 million residents in Taiwan in 2014. The NHIRD contains claims data including a registry for beneficiaries and records of diseases and other medical services. The Taiwan government renews the database every year. In the present study, information on the disease history of insured people was collected from inpatient files. The disease record system of the Taiwan NHI is based on the International Classification of Diseases, Ninth Revision, Clinical Modification (ICD-9-CM). For the safety and privacy of the insured people, the Taiwan government removes original identification numbers from the database and provides scrambled and anonymous numbers for linking the files of each insured individual before releasing the database for research purposes. The present study was approved by the Ethics Review Board of China Medical University (CMUH104-REC2-115).

### Study Population

To determine the association between alcoholic intoxication and the risk of IBD, we organized a retrospective population-based cohort study. We established 2 cohorts: an alcoholic intoxication cohort and a nonalcoholic intoxication cohort. Patients with new-onset alcoholic intoxication (ICD-9-CM codes 303, 305.0, and V113) from 1999 to 2008 were enrolled in the alcoholic intoxication cohort, and the index date was set as the date of alcoholic intoxication diagnosis. The nonalcoholic intoxication cohort comprised controls without a history of alcoholic intoxication who were randomly selected from the NHIRD; they were frequency-matched with the alcoholic intoxication patients according to age (per 5 y), sex, and index year at a ratio of 1:4. We excluded patients with a history of IBD (ICD-9-CM codes 555 and 556) before the index date. Each study patient was followed up from the index date to the year in which the patient withdrew from the NHI, an IBD event, or December 31, 2011.

We assessed the effect of alcoholic intoxication severity on the risk of IBD. We calculated the value of the total length of hospital stay owing to alcoholic intoxication divided by the total length of the follow-up duration. We categorized alcoholic intoxication severity into 3 levels according to tertiles. We also divided the subjects into 3 groups according to the admission times due to alcohol intoxication during the follow-up period.

The confounding factors of the study were age, sex, and comorbidity. A comorbidity was defined as a coexisting medical condition diagnosed during the follow-up period. The comorbidities were hypertension (ICD-9-CM codes 401–405 and 642), hyperlipidemia (ICD-9-CM code 272), diabetes mellitus (ICD-9-CM code 250), depression (ICD-9-CM codes 296.2, 296.3, 296.82, 300.4, and 311), anxiety (ICD-9-CM code 300.00), fibromyalgia (ICD-9-CM code 729.1), sleep disorder (ICD-9-CM codes 307.4 and 780.5), acute pancreatitis (ICD-9-CM code 577.0), cirrhosis (ICD-9-CM codes 571.2, 571.5, and 571.6), hepatitis B (ICD-9-CM codes V02.61, 070.20, 070.22, 070.30, and 070.32), and hepatitis C (ICD-9-CM codes V02.62, 070.41, 070.44, 070.51, and 070.54). We also considered the smoking-related illness for adjustment [6] such as stroke (ICD-9-CM: 430–438), ischemic heart disease (IHD; ICD-9-CM: 410–414), and chronic obstructive pulmonary disease (COPD; ICD-9-CM: 490–496).

### Statistical Analysis

To present the overall structure of the study cohorts, we expressed the continuous variable (age) as the mean and standard deviation (SD) and categorical variables (sex and comorbidity) as the number and percentage. To assess differences in the distributions between the 2 cohorts, the Student *t* test was used for the continuous variable, and the chi-squared test for the categorical variables. We calculated the incidence density of IBD in both cohorts. We used the Kaplan–Meier method to estimate the cumulative incidence curves for the 2 cohorts and the log-rank test to assess the curve difference. We used Cox proportional hazard regression analysis to compare crude and adjusted hazard ratios (HRs) and 95% confidence intervals (CIs) between the 2 cohorts.

All statistical analyses were conducted using the SAS 9.4 software (SAS Institute, Cary, NC, USA), and incidence curves were plotted using the R software (R Foundation for Statistical Computing, Vienna, Austria). The 2-sided level of significance was set at *P*< .05.

## Results

A total of 57 611 patients with alcoholic intoxication and 230 444 controls without alcoholic intoxication (Table 1) were enrolled in the study. Because the cohorts were frequency-matched according to age and sex, the mean age (44 y) and sex ratio (men = 90.2%) were not statistically significant between the 2 cohorts. The proportion of comorbidities was significantly greater in the alcoholic intoxication cohort than in the nonalcoholic intoxication cohort (*P*< .0001). The mean lag time between hospitalization for alcohol intoxication and the onset of IBD is 6.08 years (SD = 3.48)

**Table 1:** Comparison of demographics and history of comorbidity between alcohol intoxication and non-alcohol intoxication cohorts.

	Alcohol intoxication				
	No(N = 230 444)		Yes(N = 57 611)		
	n	%	n	%	*p*-value
**Sex**					0.99
Women	22544	9.78	5636	9.78	
Men	207900	90.2	51975	90.2	
**Age, year**					0.99
<35	54444	23.6	13611	23.6	
35–65	159740	69.3	39935	69.3	
≥65	16260	7.06	4065	7.06	
Mean (SD)^#^	44.3 (12.7)		44.3 (12.6)		0.52
**Comorbidity**					
Hypertension	3502	1.52	1296	2.25	< .0001
Hyperlipidemia	3191	1.38	1011	1.75	< .0001
Diabetes	2073	0.90	973	1.69	< .0001
Depression	1733	0.75	10394	18.0	< .0001
Anxiety	817	0.35	1877	3.26	< .0001
Fibromyalgia	717	0.31	1602	2.78	< .0001
Sleep disorder	2800	1.22	6532	11.3	< .0001
Acute pancreatitis	2150	0.93	13374	23.2	< .0001
Cirrhosis	4126	1.79	17909	31.1	< .0001
Hepatitis B	4272	1.85	6322	11.0	< .0001
Hepatitis C	2289	0.99	4345	7.54	< .0001
IHD	1999	0.87	697	1.21	< .0001
COPD	3964	1.72	1274	2.21	< .0001
Stroke	1318	0.57	802	1.39	< .0001
**Mean of follow up years (SD)**	7.62 (3.07)		6.08 (3.48)		< .0001

Chi-square test

^#^ Student’s t-test; SD, standard deviation; IHD, ischemic heart disease; COPD, chronic obstructive pulmonary disease

**Fig 1:**
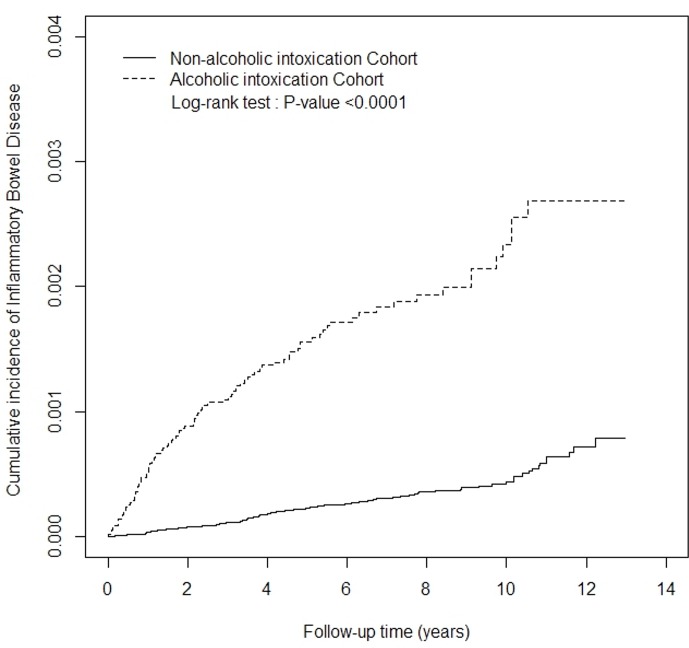
Cumulative incidence of inflammatory bowel disease. Cumulative incidence of inflammatory bowel disease in the alcoholic intoxication (dashed line) and nonalcoholic intoxication cohorts (solid line).

A total of 94 and 86 occurrences of IBD were observed in the alcoholic intoxication cohort and nonalcoholic intoxication cohort, respectively (Table 2). The incidence of IBD was 2.69 per 10 000 person-years in the alcoholic intoxication cohort and 0.49 per 10 000 person-years in the nonalcoholic intoxication cohort. Fig 1 depicts the incidence curves for the alcoholic intoxication and nonalcoholic intoxication cohorts. The results indicated that the curve for the alcoholic intoxication cohort was significantly higher than that for the nonalcoholic intoxication cohort (log-rank test, *P*< .0001) in Fig 1. After adjustment for age, sex, and comorbidity, the alcoholic intoxication cohort showed a 3.17-fold increased risk of IBD compared with the nonalcoholic intoxication cohort (HR = 3.17, 95% CI = 2.19–4.58). Compared with the nonalcoholic intoxication cohort, the HR of CD and HR of UC were 4.40 (95% CI = 2.58–7.51) and 2.33 (95% CI = 1.39–3.90) for the alcoholic intoxication cohort. Table 2 presents the results of an analysis of the incidence and adjusted HRs of IBD in the 2 cohorts after stratification according to age, sex, and comorbidity. Compared with women without alcoholic intoxication, those with alcoholic intoxication exhibited an approximately 6.75-fold (95% CI = 1.82–25.0) increased risk of IBD. The HR of IBD was only 2.90 (95% CI = 1.97–4.27) in men with alcoholic intoxication compared with those without alcoholic intoxication. The young patients with alcoholic intoxication (age <45 y) exhibited a 5.08-fold increased risk of IBD compared with those without alcoholic intoxication (HR = 5.08, 95% CI = 3.02–8.55). The old patients with alcoholic intoxication (age ≥45 y) exhibited a 1.98-fold increased risk of IBD compared with those without alcoholic intoxication (HR = 1.98, 95% CI = 1.15–3.42). The risk of IBD was significantly increased in the alcoholic intoxication patients with comorbidity (HR = 3.89, 95% CI = 2.13–7.10) and without comorbidity (HR = 3.17, 95% CI = 1.95–5.16) compared with those without alcoholic intoxication.

**Table 2:** Incidence and adjusted hazard ratio of inflammatory bowel disease stratified by sex, age and comorbidity (yes/no) between alcohol intoxication and non-alcohol intoxication cohorts.

	Alcohol intoxication						Compared to non-alcohol intoxication cohort	
		No			Yes			
Variables	Event	PY*	Rate^#^	Event	PY*	Rate^#^	IRR^‡^(95% CI)	Adjusted HR^†^(95% CI)
**Overall**	86	1756493	0.49	94	350055	2.69	5.48(5.33–5.65)***	3.17(2.19–4.58)***
Crohn disease (CD; ICD-9-CM: 555)	34	1756493	0.19	49	350055	1.40	7.23(7.01–7.46)***	4.40(2.58–7.51)***
Ulcerative colitis (UC; ICD-9-CM: 556)	52	1756493	0.3	45	350055	1.29	4.34(4.21–4.48)***	2.33(1.39–3.90)***
**Sex**								
Women	5	171353	0.29	9	37358	2.41	8.26(7.50–9.09)***	6.75(1.82–25.0)**
Men	81	1585140	0.51	85	312697	2.72	5.32(5.16–5.49)***	2.90(1.97–4.27)***
**Age, year**								
<45	34	1016697	0.33	62	210968	2.94	8.79(8.44–9.15)***	5.08(3.02–8.55)***
≥45	52	739796	0.70	32	139087	2.30	3.27(3.13–3.43)***	1.98(1.15–3.42)**
**Comorbidity**								
No	73	1603327	0.46	21	135556	1.55	3.40(3.27–3.54)***	3.17(1.95–5.16)***
Yes	13	153166	0.85	73	214500	3.40	4.01(3.67–4.38)***	3.89(2.13–7.10)***

PY*, person-year

Rate^#^, incidence rate (per 10 000 person-years)

IRR^‡^, incidence rate ratio

Adjusted HR^†^: multivariate analysis including age, sex, and comorbidities

*P < .05

**P < .01

***P < .001

Table 3 presents the risk of IBD according to the severity of alcoholic intoxication. Compared with the nonalcoholic intoxication cohort, the HRs of IBD were 1.76 (95% CI = 1.13–2.73), 6.83 (95% CI = 4.13–11.3), and 19.9 (95% CI = 12.1–32.8) for the alcoholic intoxication cohort patients with mild, moderate, and severe alcoholic intoxication, respectively. The results suggested that the risk of IBD increased with the severity of alcoholic intoxication (*p* for the trend < .0001) by the Cox proportional hazard regression model. Table 3 also displays the incidence rate of IBD (per 10,000 person-years) increased with hospitalization times, from 2.34 in 1 time of hospitalization, to 3.18 in 2 times, and 3.87 in more than or equal to 3 times. While after adjustment with age, sex, and comorbidities, the adjusted HRs for the three groups are 3.15, 3.34, 3.09, respectively, with the highest HR in patients with 2 times of hospitalization. Nonetheless, the trend is still significant by the Cox proportional hazard regression model (*p* for trend < .0001).

**Table 3:** Incidence rate and hazard ratio for inflammatory bowel disease stratified by severity of alcohol intoxication.

Severity of alcohol intoxication	Event	Rate	Adjusted HR^†^(95% CI)
**Non-alcohol intoxication cohort**	86	0.49	Ref
**Alcohol intoxication cohort**			
Mild (T_1_)	33	1.21	1.76(1.13–2.73)**
Moderate (T_2_)	28	5.12	6.83(4.13–11.3)***
Severe (T_3_)	33	14.14	19.9(12.1–32.8)***
*p* for trend^*#*^			< .0001
**Number of hospitalizations due to alcohol intoxication during the follow-up time**			
1	58	2.34	3.15(2.16–4.59)***
2	16	3.18	3.34(1.82–6.12)***
≥3	20	3.87	3.09(1.62–5.89)***
*p* for trend^*#*^			< .0001

Rate, incidence rate, per 10 000 person-years; HR, adjusted hazard ratio; CI, confidence interval; T_1_, first tertile; T_2_, second tertile; T_3_, third tertile. Severity of alcoholism = (total length of hospital stay due to alcohol intoxication during the follow-up duration) ÷ (length of follow-up duration).

^†^Adjusted for age, sex, and each comorbidities.

^#^ The p for trend was estimated by the Cox proportional hazard regression model.

**P < .01

***P < .001

## Discussion

Among the environmental factors identified to influence the risk of IBD, cigarette smoking is the most extensively studied. Cigarette smoking habit was indicated that it is not an attributable risk factor for UC and may have an inverse effect on the development of UC. However, the previous study was present that cigarette smoking habit might be associated with an increased risk for CD [7]. Alcoholic intoxication, or hazardous and harmful alcohol use, is defined as the daily consumption of >40 g of alcohol in men and 20 g in women, as another important environmental factor for chronic illness [8]. However, the effect of alcoholic intoxication on CD and UC remains little known and not studied widely.

Several studies have investigated the relationship between alcohol consumption and IBD; however, the results are inconsistent. Two previous studies have observed that alcohol consumption frequently worsens GI symptoms in patients with IBD [9, 10]. Three studies on patients with CD have demonstrated contradictory outcomes; one study reported that alcohol may have a reduced risk for CD [11], and another observed an increased alcohol intake in patients recently diagnosed with CD compared with healthy controls [12]. The other study reported that alcohol has no effect on CD [13]. Such contradictory results were also noticed in 4 studies on the relationship between alcohol and UC [14–17]. The possible main problem resulting in these contradictory results is that most studies do not assess the frequency and amount of alcohol ingestion, and thus, the alcohol consumption group may comprise dissimilar proportions of patients with alcoholic intoxication.

Two studies on UC that have recorded the frequency and amount of alcohol consumption have obtained more detailed findings. Jiang et al reported that light alcohol drinking before diagnosis has a protective effect on UC development (OR = 0.52, 95% CI = 0.32–0.85, *P* = .009) [18]. Jowett et al reported that alcohol in the top tertile of intake increases the likelihood of relapse compared with that at the bottom tertile of intake (OR = 2.71, 95% CI = 1.1–6.67). In that cohort, patients who had a relapse or disease flare over 1 year consumed 14 g of alcohol daily compared with 10 g daily in those who had no relapse [19]. Swanson et al discovered that patients with inactive IBD who consumed moderate red wine daily (approximately 0.4 g of alcohol/kg of body weight) are associated with a significant decrease in stool calprotectin and a significant increase in intestinal permeability, which made them at an increased long-term risk of disease relapse [20].

Grønbæk et al reported that alcoholic cirrhosis increased the risk of IBD in a nationwide cohort study (adjusted incidence rate ratio = 1.56; 95% CI = 1.26–1.92) [21]. Our data may in part answer the question about the existence of the risk of IBD before cirrhosis is developed. In present study, the risk was observed even after excluding patients with cirrhosis and other comorbidities.

The results of the present and previous studies suggest that light alcoholic drinking may have a protective effect against IBD, whereas moderate-to-heavy alcohol drinking increases the risk of IBD. This phenomenon was observed in patients with and without alcoholic cirrhosis.

Alcohol may exert a protective effect by inhibiting the systemic immune system and neutrophil migration [22]. Furthermore, some antioxidants present in red wine, such as resveratrol, have anti-inflammatory properties [23], and thus, light alcohol drinking can potentially prevent the relapse of IBD. By contrast, alcohol can increase the activity of liver Kupffer cells and thus increase the production of pro-inflammatory mediators such as tumor necrosis factor-α, interleukin-1 (IL-1), and IL-6 [24]. Moreover, alcohol consumption can disrupt gut barrier function and increase intestinal permeability [25], to which patients with IBD are particularly vulnerable [20, 26]. Another possible threat is that some alcoholic beverages cause osmotic diarrhea because of the high sugar content [27]. In addition, alcoholic drinks are rich sources of sulfur and sulfate, which increase the concentration of fecal hydrogen sulfide, which is toxic to colonocytes [19]. In the case of moderate-to-heavy drinking, these deleterious effects of alcohol may be exaggerated and overweigh its benefit.

This study used the NHIRD claims data and has certain limitations. 1) The NHI database does not disclose the personal history of patients, including the family history, smoking habits, diet, occupational exposure, and family disease history. Past studies estimated that over 83% of alcoholics also smoke, with alcoholism approximately 10 times more prevalent in smokers than in non-smokers [28, 29]. A national health survey in Taiwan reported that 81.1% of smokers had alcohol consumption habit [30]. Therefore, a large proportion of subjects in the alcohol intoxication group are possible smokers. Because we had no smoking history data and the relationship of smoking and IBD has been correlated, we have replaced it with the comorbidity data associated with smoking. According to Yeh CC et al [6], we have added stroke (ICD-9-CM: 430–438), ischemic heart disease (IHD; ICD-9-CM: 410–414), and chronic obstructive pulmonary disease (COPD; ICD-9-CM: 490–496) for adjustment. Cigarette smoking is well known for its different effects on CD and UC. Smoking is associated with an increased risk of CD (HR = 1.46 to 1.9) [31, 32] but may be protective of the development and relapse of UC (HR = 0.63 to 0.8) [33, 34]. In our study, the adjusted HRs of alcohol intoxication for CD and UC are 4.40(2.58–7.51) and 2.33(1.39–3.90), respectively. Even if we hypothesize that all subjects in the alcohol intoxication group are smokers and those in the control group are all non-smokers, after dividing by the HR of smoking, the HRs of alcohol intoxication will become 2.32 to 3.01 for CD and 2.91 to 3.70 for UC, respectively. 2) Diagnostic accuracy depends on the performance of clinical physicians. Furthermore, the frequency of alcohol consumption, amount of daily intake, and type of alcoholic beverages were not available in the claims database. However, we pointed out that the patients with alcohol intoxication as severe alcohol-related impact and extremely high alcohol consumption. The risks for IBD would be possibly underestimated among the hidden and/or slight alcohol consumption population, but the estimation of risk for IBD would present the bias toward the null following alcohol intoxication population. We considered that the observation of alcohol intoxication population via population study would be existed importance for preventive and health political decision among the alcohol-related health impacts, especially in IBD.

## Conclusion

In conclusion, by analyzing nationwide health insurance data, we demonstrated that alcoholic intoxication was associated with a 3.17-fold increased risk of IBD after adjustment for age, sex, and comorbidities. The risk for IBD increased with the length of hospital stay because of alcoholic intoxication as a significant gradient response. The severity of alcohol intoxication might be correlated with duration, like length of hospital stay, and we suggested that the physicians should pay attention to the alcohol intoxication patients with long-term or recurrent hospitalization.

## Supporting Information

S1 Checklist RECORD checklist for the Reporting of observational studies. (DOCX)

## Abbreviations

AI: alcoholic intoxication
CD: Crohn disease
CI: confidence interval
COPD: chronic obstructive pulmonary disease
GI: gastrointestinal
HR: hazard ratio
IBD: inflammatory bowel disease
ICD-9- CM: International Classification of Diseases, Ninth Revision, Clinical Modification
IHD: ischemic heart disease
IL-1: interleukin-1
NHI: National Health Insurance
NHIRD: National Health Insurance Research Database
SD: standard deviation
UC: ulcerative colitis
