# Supplementary material for: Effect of Alcoholic Intoxication on the Risk of Inflammatory Bowel Disease: A Nationwide Retrospective Cohort Study
Source: PLoS One. 2016 Nov 1;11(11):e0165411. doi: 10.1371/journal.pone.0165411 (PMC5089729; doi:10.1371/journal.pone.0165411)
Supplement: S1 Checklist — (DOCX) [file pone.0165411.s001.docx]

**S1 Checklist. RECORD checklist for the Reporting of observational studies.**

The RECORD statement – checklist of items, extended from the STROBE statement, that should be reported in observational studies using routinely collected health data.

|  | **Item No.** | **STROBE items** | **Location in manuscript where items are reported** | **RECORD items** | **Location in manuscript where items are reported** |
| --- | --- | --- | --- | --- | --- |
| **Title and abstract** | | | | | |
|  | 1 | (a) Indicate the study’s design with a commonly used term in the title or the abstract (b) Provide in the abstract an informative and balanced summary of what was done and what was found | (a)Title “**Effect of Alcoholic intoxication on the Risk of Inflammatory Bowel Disease: A Nationwide Retrospective Cohort Study**”  (b)Abstract: Purpose: This study investigated whether alcoholic intoxication (AI) increases the risk of inflammatory bowel disease (IBD) by using a population-based database in Taiwan.  Methods: This retrospective matched-cohort study included 57 611 inpatients with new-onset AI (AI cohort) and 230 444 randomly selected controls (non-AI cohort). Each patient was monitored for 10 years to individually identify those who were subsequently diagnosed with Crohn disease (CD) and ulcerative colitis (UC) during the follow-up period. Cox proportional hazard regression analysis was conducted to determine the risk of IBD in patients with AI compared with controls without AI.  Results: The incidence rate of IBD during the 10-year follow-up period was 2.69 per 1 000 person-years and 0.49 per 1 000 person-years in the AI and non-AI cohorts, respectively. After adjustment for age, sex, and comorbidity, the AI cohort exhibited a 3.17-fold increased risk of IBD compared with the non-AI cohort (hazard ratio [HR] = 3.17, 95% confidence interval [CI] = 2.19 - 4.58). Compared with the non-AI cohort, the HRs of CD and UC were 4.40 and 2.33 for the AI cohort, respectively. After stratification for the severity of AI according to the duration of hospital stay, the adjusted HRs exhibited a significant correlation with the severity; the HRs of IBD were 1.76, 6.83, and 19.9 for patients with mild, moderate, and severe AI, respectively (p for the trend < .0001).  Conclusion: The risk of IBD was higher in patients with AI and increased with the length of hospital stay. | RECORD 1.1: The type of data used should be specified in the title or abstract. When possible, the name of the databases used should be included.  RECORD 1.2: If applicable, the geographic region and timeframe within which the study took place should be reported in the title or abstract.  RECORD 1.3: If linkage between databases was conducted for the study, this should be clearly stated in the title or abstract. | RECORD 1.1: Title “**Effect of Alcoholic intoxication on the Risk of Inflammatory Bowel Disease: A Nationwide Retrospective Cohort Study**”  RECORD 1.2: N/A  RECORD 1.3:  Abstract: Purpose: This study investigated whether alcoholic intoxication (AI) increases the risk of inflammatory bowel disease (IBD) by using a population-based database in Taiwan.  Methods: This retrospective matched-cohort study included 57 611 inpatients with new-onset AI (AI cohort) and 230 444 randomly selected controls (non-AI cohort). Each patient was monitored for 10 years to individually identify those who were subsequently diagnosed with Crohn disease (CD) and ulcerative colitis (UC) during the follow-up period. Cox proportional hazard regression analysis was conducted to determine the risk of IBD in patients with AI compared with controls without AI.  Results: The incidence rate of IBD during the 10-year follow-up period was 2.69 per 1 000 person-years and 0.49 per 1 000 person-years in the AI and non-AI cohorts, respectively. After adjustment for age, sex, and comorbidity, the AI cohort exhibited a 3.17-fold increased risk of IBD compared with the non-AI cohort (hazard ratio [HR] = 3.17, 95% confidence interval [CI] = 2.19 - 4.58). Compared with the non-AI cohort, the HRs of CD and UC were 4.40 and 2.33 for the AI cohort, respectively. After stratification for the severity of AI according to the duration of hospital stay, the adjusted HRs exhibited a significant correlation with the severity; the HRs of IBD were 1.76, 6.83, and 19.9 for patients with mild, moderate, and severe AI, respectively (p for the trend < .0001).  Conclusion: The risk of IBD was higher in patients with AI and increased with the length of hospital stay. |
| **Introduction** | | | | | |
| Background rationale | 2 | Explain the scientific background and rationale for the investigation being reported | Introduction, page 7-8 |  |  |
| Objectives | 3 | State specific objectives, including any prespecified hypotheses | Introduction, page 7-8 |  |  |
| **Methods** | | | | | |
| Study Design | 4 | Present key elements of study design early in the paper | Materials and Methods, Part of “Study Population” , Page 9-10 |  |  |
| Setting | 5 | Describe the setting, locations, and relevant dates, including periods of recruitment, exposure, follow-up, and data collection | Materials and Methods, Part of “Data Source” and “Study Population ", Page 9-10 |  |  |
| Participants | 6 | *(a) Cohort study* - Give the eligibility criteria, and the sources and methods of selection of participants. Describe methods of follow-up  *Case-control study* - Give the eligibility criteria, and the sources and methods of case ascertainment and control selection. Give the rationale for the choice of cases and controls  *Cross-sectional study* - Give the eligibility criteria, and the sources and methods of selection of participants  *(b) Cohort study* - For matched studies, give matching criteria and number of exposed and unexposed  *Case-control study* - For matched studies, give matching criteria and the number of controls per case | 1. Materials and Methods, Part of “Study Population ", Page 9-10 2. Materials and Methods, Part of “Study Population ", Page 9-10 | RECORD 6.1: The methods of study population selection (such as codes or algorithms used to identify subjects) should be listed in detail. If this is not possible, an explanation should be provided.  RECORD 6.2: Any validation studies of the codes or algorithms used to select the population should be referenced. If validation was conducted for this study and not published elsewhere, detailed methods and results should be provided.  RECORD 6.3: If the study involved linkage of databases, consider use of a flow diagram or other graphical display to demonstrate the data linkage process, including the number of individuals with linked data at each stage. | RECORD 6.1: Materials and Methods, Part of “Study Population ", Page 9-10  RECORD 6.2: None  RECORD 6.3: none |
| Variables | 7 | Clearly define all outcomes, exposures, predictors, potential confounders, and effect modifiers. Give diagnostic criteria, if applicable. | Materials and Methods, Part of “Study Population” and “Statistical Analysis”, Page 9-11 | RECORD 7.1: A complete list of codes and algorithms used to classify exposures, outcomes, confounders, and effect modifiers should be provided. If these cannot be reported, an explanation should be provided. | RECORD 7.1:  Materials and Methods, Part of “Study Population” and “Statistical Analysis” , Page 9-11 |
| Data sources/ measurement | 8 | For each variable of interest, give sources of data and details of methods of assessment (measurement).  Describe comparability of assessment methods if there is more than one group | Materials and Methods, Part of “Study Population” and “Statistical Analysis” , Page 9-11 |  |  |
| Bias | 9 | Describe any efforts to address potential sources of bias | Materials and Methods, Part of “Data Source” , Page 8 |  |  |
| Study size | 10 | Explain how the study size was arrived at | Materials and Methods, Part of “Study Population” , Page 9 |  |  |
| Quantitative variables | 11 | Explain how quantitative variables were handled in the analyses. If applicable, describe which groupings were chosen, and why | Materials and Methods, Part of “Study Population” and “Statistical Analysis”, Page 9-11 |  |  |
| Statistical methods | 12 | (a) Describe all statistical methods, including those used to control for confounding  (b) Describe any methods used to examine subgroups and interactions  (c) Explain how missing data were addressed  (d) *Cohort study* - If applicable, explain how loss to follow-up was addressed  *Case-control study* - If applicable, explain how matching of cases and controls was addressed  *Cross-sectional study* - If applicable, describe analytical methods taking account of sampling strategy  (e) Describe any sensitivity analyses | (a)Materials and Methods, Part of “Study Population” and “Statistical Analysis”, Page 9-11  (b) Materials and Methods, “Statistical Analysis”, Page 11  (c)None  (d) Materials and Methods, Part of “Study Population” and “Statistical Analysis”, Page 9-11 |  |  |
| Data access and cleaning methods |  | .. |  | RECORD 12.1: Authors should describe the extent to which the investigators had access to the database population used to create the study population.  RECORD 12.2: Authors should provide information on the data cleaning methods used in the study. | RECORD 12.1:  Materials and Methods, Part of “Study Population” and “Statistical Analysis” , Page 9-11  RECORD 12.2: |
| Linkage |  | .. |  | RECORD 12.3: State whether the study included person-level, institutional-level, or other data linkage across two or more databases. The methods of linkage and methods of linkage quality evaluation should be provided. | Materials and Methods, Part of “Data Source”, Page 8-9  RECORD 12.3:  None |
| **Results** | | | | | |
| Participants | 13 | (a) Report the numbers of individuals at each stage of the study (*e.g.*, numbers potentially eligible, examined for eligibility, confirmed eligible, included in the study, completing follow-up, and analysed)  (b) Give reasons for non-participation at each stage.  (c) Consider use of a flow diagram | (a)Result, first Paragraph.  (b) Materials and Methods, Part of “Data Source”, Page 8-9  (c) none | RECORD 13.1: Describe in detail the selection of the persons included in the study (*i.e.,* study population selection) including filtering based on data quality, data availability and linkage. The selection of included persons can be described in the text and/or by means of the study flow diagram. | RECORD 13.1: Result, first Paragraph. |
| Descriptive data | 14 | (a) Give characteristics of study participants (*e.g.*, demographic, clinical, social) and information on exposures and potential confounders  (b) Indicate the number of participants with missing data for each variable of interest  (c) *Cohort study* - summarise follow-up time (*e.g.*, average and total amount) | (a)Result, first Paragraph.  (b)n/a  (c)Table 1 |  |  |
| Outcome data | 15 | *Cohort study* - Report numbers of outcome events or summary measures over time  *Case-control study* - Report numbers in each exposure category, or summary measures of exposure  *Cross-sectional study* - Report numbers of outcome events or summary measures | Result, first Paragraph. |  |  |
| Main results | 16 | (a) Give unadjusted estimates and, if applicable, confounder-adjusted estimates and their precision (e.g., 95% confidence interval). Make clear which confounders were adjusted for and why they were included  (b) Report category boundaries when continuous variables were categorized  (c) If relevant, consider translating estimates of relative risk into absolute risk for a meaningful time period | (a) Result, Second Paragraph, and Table 2  (b)Result, first Paragraph, and Table 1  (c) Result, Second Paragraph. |  |  |
| Other analyses | 17 | Report other analyses done—e.g., analyses of subgroups and interactions, and sensitivity analyses | Result, fourth Paragraph, and Table 3. |  |  |
| **Discussion** | | | | | |
| Key results | 18 | Summarise key results with reference to study objectives | Discussion, First Paragraph, first three sentence. |  |  |
| Limitations | 19 | Discuss limitations of the study, taking into account sources of potential bias or imprecision. Discuss both direction and magnitude of any potential bias | Discussion, Last Paragraph. | RECORD 19.1: Discuss the implications of using data that were not created or collected to answer the specific research question(s). Include discussion of misclassification bias, unmeasured confounding, missing data, and changing eligibility over time, as they pertain to the study being reported. | RECORD 19.1:  Descripted as left column. |
| Interpretation | 20 | Give a cautious overall interpretation of results considering objectives, limitations, multiplicity of analyses, results from similar studies, and other relevant evidence | Discussion, last paragraph. |  |  |
| Generalisability | 21 | Discuss the generalisability (external validity) of the study results | Discussion, Second Paragraph |  |  |
| **Other Information** | | | | | |
| Funding | 22 | Give the source of funding and the role of the funders for the present study and, if applicable, for the original study on which the present article is based | “The Management Office for Health Data, China Medical University Hospital, Taichung, Taiwan is supported in part by Taiwan Ministry of Health and Welfare Clinical Trial and Research Center of Excellence (MOHW105-TDU-B-212-133019), China Medical University Hospital, Academia Sinica Taiwan Biobank Stroke Biosignature Project (BM10501010037), NRPB Stroke Clinical Trial Consortium (MOST 104-2325-B-039 -005), Tseng-Lien Lin Foundation, Taichung, Taiwan, Taiwan Brain Disease Foundation, Taipei, Taiwan, and Katsuzo and Kiyo Aoshima Memorial Funds, Japan; and CMU under the Aim for Top University Plan of the Ministry of Education, Taiwan. No additional external funding was received for this  study. The funders had no role in study design, data collection and analysis, decision to publish, or preparation of the manuscript.” |  |  |
| Accessibility of protocol, raw data, and programming code |  | .. |  | RECORD 22.1: Authors should provide information on how to access any supplemental information such as the study protocol, raw data, or programming code. | RECORD 22.1:  The data on the study population that were obtained from the NHIRD  (http://nhird.nhri.org.tw/en/index.html) are maintained in the NHIRD  (<http://nhird.nhri.org.tw/)>, Accessed 2016 Oct 14 |

*Reference: Benchimol EI, Smeeth L, Guttmann A, Harron K, Moher D, Petersen I, Sørensen HT, von Elm E, Langan SM, the RECORD Working Committee. The REporting of studies Conducted using Observational Routinely-collected health Data (RECORD) Statement. *PLoS Medicine* 2015; in press.

*Checklist is protected under Creative Commons Attribution ([CC BY](http://creativecommons.org/licenses/by/4.0/)) license.
